# Development of a Feature and Template-Assisted Assembler and Application to the Analysis of a Foot-and-Mouth Disease Virus Genotyping Microarray

**DOI:** 10.1371/journal.pone.0166870

**Published:** 2017-01-03

**Authors:** Roger W. Barrette, Jessica M. Rowland, Frederic R. Grau, Michael T. McIntosh

**Affiliations:** 1 United States Department of Agriculture, Animal and Plant Health Inspection Service, Foreign Animal Disease Diagnostic Laboratory, Plum Island Animal Disease Center, Orient, NY, United States of America; 2 Department of Environmental and Global Health, Emerging Pathogens Institute University of Florida, Gainesville, FL, United States of America; The Scripps Research Institute, UNITED STATES

## Abstract

Several RT-PCR and genome sequencing strategies exist for the resolution of Foot-and-Mouth Disease virus (FMDV). While these approaches are relatively straightforward, they can be vulnerable to failure due to the unpredictable nature of FMDV genome sequence variations. Sequence independent single primer amplification (SISPA) followed by genotyping microarray offers an attractive unbiased approach to FMDV characterization. Here we describe a custom FMDV microarray and a companion feature and template-assisted assembler software (FAT-assembler) capable of resolving virus genome sequence using a moderate number of conserved microarray features. The results demonstrate that this approach may be used to rapidly characterize naturally occurring FMDV as well as an engineered chimeric strain of FMDV. The FAT-assembler, while applied to resolving FMDV genomes, represents a new bioinformatics approach that should be broadly applicable to interpreting microarray genotyping data for other viruses or target organisms.

## Introduction

FMD is a highly contagious vesicular disease affecting many species of wild and domestic cloven-hoofed ungulates including livestock such as captive cervids, sheep, goats, pigs, buffalo and cattle. Early detection and effective outbreak control are critical to limiting economic losses associated with FMD outbreaks. FMD is caused by the FMD virus (FMDV), a small positive-sense RNA virus belonging to the genus Aphthovirus within the family *Picornaviridae*. FMDV are characteristically heterogeneous comprising multiple different topotypes within any of 7 different serotypes. Rapid phylogenetic characterization is critical to appropriate vaccine selection as part of an outbreak control or recovery strategy.

Microarray-based methods to identify and characterize pathogens have been described for many years. While originally developed for identification of single nucleotide polymorphisms (SNPs) [1,2], development of higher density microarrays has enabled increased resolution to include resequencing of pathogen genes [3]. The capacity of microarrays to resequence large genome regions of rapidly evolving RNA viruses is limited, as the number of features required to represent all potential genetic variations can exceed even the highest density microarray formats. However, recent advances in bioinformatics and scripting tools allow for streamlined development of analysis methods to enhance the resolution of pathogen characterization and resequencing.

While next-generation sequencing (NGS) is increasingly employed in unbiased pathogen detection and genotyping, high equipment costs and the need for specialized sequence analysis expertise continue to limit its use in routine genotyping of viruses for some laboratories. Additionally, preparation of DNA libraries for NGS can be time consuming, and is highly dependent on the quality of sample nucleic acid.

A need for robust genotyping methods that are not critically impacted by genetic polymorphisms has resulted in the development of genotyping and serotyping microarrays for a variety of pathogens [3–5]. Combined with random nucleic acid amplification methods, microarrays can perform unbiased parallel analyses in a single hybridization making them powerful pathogen detection tools [4,6–8]. In addition, the availability of multi-format, high density microarrays allows for high-throughput applications. Unlike directed PCR genotyping or gene-specific sequencing, genotyping microarrays are not dependent upon the sequence of a specific pair of primers but rather rely on patterns of reactive microarray features from a large pool of features unique to various known genotypes and isolates. For example, microarrays capable of detecting highly polymorphic foot-and-mouth disease virus (FMDV) or other vesicular disease viruses have been developed and proven effective. [9–11]

In this paper, we describe our Feature and Template-assisted Assembler (FAT-assembler) which is a new bioinformatics approach to resolve virus genome sequence using only a moderate number of conserved microarray features. This approach allows for a fewer number of microarray features with the aim of reducing costs and increasing throughput of microarray-based resequencing or genotyping and can enable the coupling of resequencing and pathogen detection arrays. The algorithm combines sequence data from microarray features, their position within a viral genome, and the intensity of signal for each feature observed during analysis to produce ‘high-resolution’ interpretive sequence data from a virus-specific genotyping microarray. Applied to the analysis of an FMDV genotyping microarray, the approach enabled rapid characterization of virus serotypes and topotypes relevant for epidemiological or forensic trace-back, or as a guide to vaccine matching. It is hoped that application of this approach to the analysis of resequencing microarrays may enable the development of smaller more cost-effective microarrays along with improved speed and confidence in genotyping results.

There is an ongoing need for methods of rapid identification of viruses during outbreaks to help mount an adequate response for limiting the spread of disease. FMDV, for instance, is of great concern worldwide due to its broad impact on agriculture and international trade. Rapid and accurate characterization of FMDV is needed for selection of appropriate vaccines due to the lack of cross-protection between various virus serotypes and topotypes. This involves differentiation into one of the many sub-types, also known as topotypes, within each serotype. Further complicating the identification and characterization of FMDV is the error-prone viral RNA polymerase that results in a large population of quasispecies [12][13] during infection. This high level of genetic drift requires that genome sequence characterization be performed to accurately identify the specific isolate. This makes this particular virus an excellent model candidate for testing this type of analysis.

## Methods

### Microarray Design

The 3 kb capsid coding (P1) regions for each of the seven known serotypes of FMDV were selected from the NCBI Genbank nucleotide database. FMDV P1 coding sequences were then fragmented into overlapping 23-25mers, and Tm predictions were made based on the nearest-neighbor method (Sambrook and Russell, 2001). The complete list of features was then consolidated to eliminate redundancy. Microarray features were submitted to Agilent (Agilent Technologies) using the eArray interface for 8x60K custom microarray synthesis.

### Nucleic Acid Amplification

Nucleic acids were extracted from tissue culture isolates of FMDV using the RNeasy® Mini Kit (Qiagen Inc.). Reverse transcription PCR (RT-PCR) was performed on 10 μl of total RNA using 1 μl (200 U/ μl) SuperScript III (Life Technologies) in a 40 μl reaction containing 10 μM tagged random primer (*GTTTCCAAGTCACGATC*NNNNNNNNN) and 10 μM tagged oligo-dT primer (*GTTTCCAAGTCACGATC*TTTTTTTTTTTT). The cDNA product was treated with 2 units of RNAse H (Life Technologies ™) at 37°C for 30 min and purified by QIAquick® PCR Purification Kit (Qiagen Inc.). Tagged cDNA (5 μl) was denatured for 3 min at 95°C, and rapidly cooled on ice for 2 min before being treated with 15 units of terminal deoxynucleotidyl transferase (TdT, Life Technologies) in 5 μl of tailing buffer containing 50 mM TrisCl pH 8.4, 125 mM KCl, 7.5 mM MgCl2, and 0.2 mM dATP for 10 min at 37°C followed by 65°C for 10 min. Tailed cDNA was repurified by QIAquick® PCR Purification Kit (Qiagen, Inc.). PCR was then performed in a 100 μl reaction using Platinum® PCR Supermix (Life Technologies™), 8 μl tailed cDNA, 10 μM Tag primer (*GTTTCCAAGTCACGATC*) and 10 μM Tagged oligo-dT primer (described above). PCR was performed using a Tetrad thermocycler (Bio-Rad) with an initial 8 min 95°C denature, followed by 30 cycles of 95°C for 30 sec, 40°C for 30 sec, 50°C for 30 sec, and 72°C for 60 sec. The final PCR product was purified using the QIAquick® PCR Purification Kit (Qiagen, Inc.).

### Nucleic Acid Labeling, Array Hybridization and Washing

Purified PCR product was labeled using BioPrime® Array CGH Genomic Labeling System (Life Technologies) with Alexa Fluor® 555 (Molecular Probes) and purified by passing through a NucAway™ column (Ambion). Fluorescently labeled PCR products were concentrated by dehydration in a Thermo Scientific Savant SpeedVac and reconstituted in nuclease-free water to a final volume of 16 μl. Hybridization and washing of Agilent arrays was performed in Agilent HI-RPM hybridization and blocking buffer for 16 hr at 57°C in an Agilent rotating hybridization incubator. After hybridization, arrays were washed at room temperature for 5 min in 200 ml of Agilent CGH buffer 1 with mixing. Arrays were then transferred directly to another slide tray containing 200 ml of Agilent CGH wash buffer 2 and incubated at 37°C for 1 min. Finally arrays were transferred to a phosphate buffered saline solution at pH 8.0 (Sigma Aldrich) and incubated with mixing for 5 min, dried by centrifugation at 700 x g for 1 min in a 50 ml conical tube with a Kimwipe inserted into the bottom of the tube to act as a wick. Upon completion of drying, slides were scanned and analyzed for fluorescence intensity of features.

### Scanning and Analysis

Microarray scanning was performed using a GenePix® 4400A at 532 nm wavelength using a standard green filter, 3 line averaging, and 5 μM resolution. GenePix® Pro 7.1 was used for data capture. Data analysis was performed using programs written in the Python programming language (Python 2.7.5, Python Software Foundation).

### Method for Inferring Sequence Template using FAT-Assembler

#### Microarray data parsing and selection of positive features

**Fig 1:**
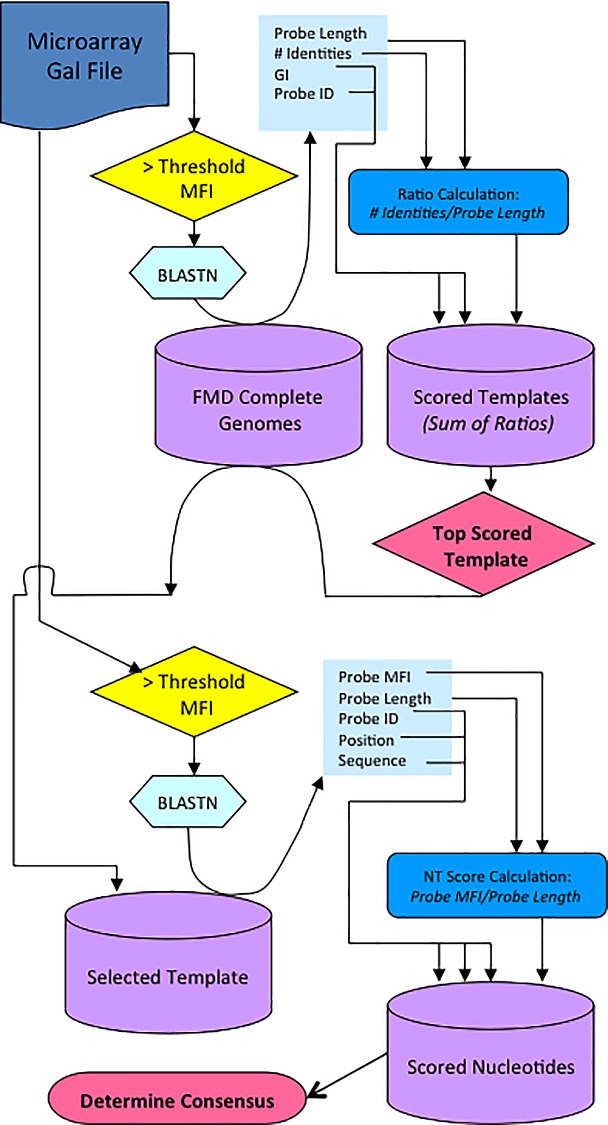
Flowchart illustrating the scoring process for determining sequence by microarray. Process begins with the scan results file (.gal) to extract MFI data for the purpose of determining the best template sequence and the downstream consensus. A. Features with MFI that are greater than the threshold are initially evaluated by a series of local BLAST alignments to a database of complete FMDV genomes to assemble a genome ‘scaffold’. B. Once the scaffold is produced, the process is repeated with the scaffold sequence as the reference, and individual nucleotides are scored and selected for determination of the final consensus sequence.

Data from microarray analysis was used as input for the algorithm (Fig 1) which performs a series of template selections of sequential overlapping positive microarray sequence features. These sequence fragments are then assembled into a final template that is used to determine the sequence of a given FMDV. Initially mean fluorescence intensity (MFI) data is selected from the microarray output file (.GPR) by a simple MFI threshold of >2000 MFI at 532nm wavelength. Features determined to be ‘positive’ by this cutoff are recorded into a MySQL database, along with their corresponding feature sequence. Feature sequence data is then recovered from the database and initially copied into an intermediate FASTA formatted file that is subsequently converted into a BLAST database using the formatdb tool (NCBI).

#### Sequence template selection

Viral genomes may display genetic recombination in addition to genetic drift making selection of a given reference genome a potentially poor or misleading choice for use as a template with which to compare microarray features for resequencing of a new strain of virus. To arrive at the best possible choice for a template sequence for resequencing, a FASTA formatted alignment file is generated to include overlapping 500 nucleotide sequences spanning the P1 coding regions for all known FMDV genomes present in the NCBI Genbank database. The overlapping reference sequences are iteratively compared against each ‘positive’ selected feature sequence within microarray results database. At each overlapping 500 bp position, the highest scoring segment against the positive data set is recorded in a FASTA formatted file. Upon completion of the template selection, the FASTA file containing the sequential fragments are passed through the consensus assembly software, ‘CAP2’; with threshold parameters set to 20% overlap and 80% similarity for performing alignments to produce a full consensus template sequence. The output of this assembly provides an optimized template from which sequence assembly is ultimately determined.

#### Template-based sequence assembly

Upon assembly of the optimized FMDV reference template, the FASTA output file is converted into a BLAST database using makeblastdb. Data from the microarray output file is filtered based on a logarithmically increasing MFI threshold, and sequence assembly is performed at each of these cutoffs (2000, 4000, 8000, 16000, 32000, and 64000). Corresponding positive microarray feature sequences are compared to the optimized reference template using BLASTN to determine the best feature matches and probe positions against the template. Results from the BLAST analysis are stored in a separate table within the database for downstream nucleotide positional scoring analysis.

#### Nucleotide positional scoring

Nucleotide positional scoring was performed by dividing the total sum of the MFI of each feature by its length in nucleotides.

∑(MFI/Probe_Length)

If more than one possible nucleotide is predicted to occur at a given position, then the nucleotide with the highest score is selected. Selected nucleotides at each position are used to assemble an intermediate consensus sequence for each of the described MFI thresholds.

#### Determination of final consensus sequence

**Fig 2:**
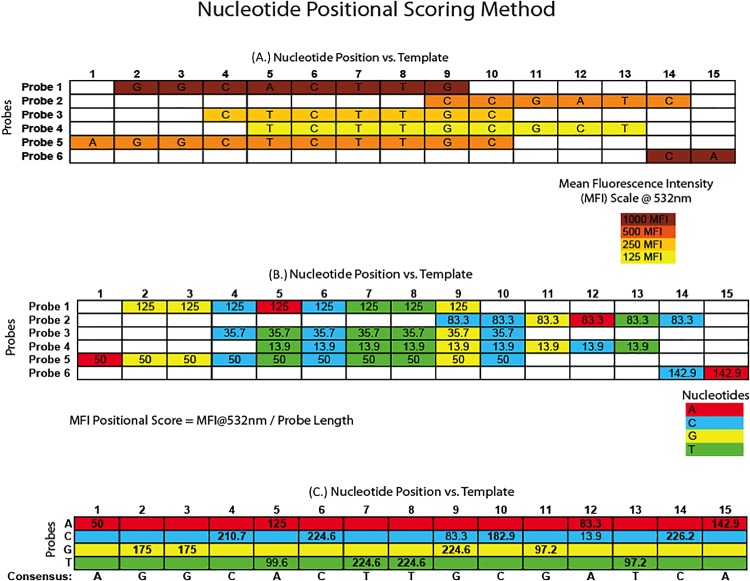
Calculations for inferring nucleotide identities at each position compared to a template sequence. **2A. Microarray feature nucleotide alignment.** This represents an alignment of candidate feature sequences in relation to the reference scaffold with the individual nucleotides at each position**; 2B. Mean-fluorescent intensity scoring of alignment.** These are the weighted scores calculated by determining the MFI of the feature divided by the length of the probe to determine the individual nucleotide score. Different colors represent each of four individual nucleotides.**; 2C. Final scoring and nucleotide selection.** This chart shows sum scores for each of the possible nucleotides for each position of the alignment, with the highest scoring nucleotide being selected for the downstream consensus sequence. Consensus sequences are produced for multiple MFI thresholds.

Each of the assembled intermediate consensus sequences from each of the MFI thresholds are compared to each other by evaluating each individual nucleotide position. At each position, the final selected nucleotide is determined by the greatest MFI threshold. This is repeated, for example, at approximately 3000 nucleotide positions representing the complete P1 coding region of the FMDV genome (Fig 2).

### Computer Programs and Algorithms

All modules of the algorithm were assembled in Python (version 2.5.1). Database management was controlled through Python, using a MySQL (version 5.5) database server for data storage. BLAST analysis and searchable database assembly was performed using NCBI bioinformatics tools (version 20070822).

## Results and Discussion

The ability to perform rapid genotyping of pathogens is a very useful tool during outbreaks. Having bioinformatics tools which can extract sequence data from sequence based datasets such as genotyping microarrays offers the advantage of resolving data at a higher resolutions than simple feature origin SNP arrays or SNP-specific PCR assays. Here we describe an analysis algorithm that works with an FMD genotyping array for inferring sequence of the P1 region of FMDV; although this method could easily be adapted to other targeted arrays or NGS data. In the event of an outbreak of FMD with high degrees of genomic polymorphism between and within virus species, rapid and accurate genetic characterization will be essential to vaccine matching studies. As the density of features on microarrays continues to increase, the potential for improved identification will also expand. However, even with high-density microarrays, efficient algorithms for feature selection and analysis are still necessary and will further increase the power of these platforms. Here we describe an algorithm for deriving interpretive sequence via an intermediate scaffold using relatively low density microarray data from a genotyping microarray specific to FMDV as model data for this analysis software.

### Characterization of Seven Serotypes of FMDV

Seven isolates of FMDV representing each of the known serotypes (A, Asia 1, O, C, SAT1, SAT2, and SAT3) were tested using a genotyping microarray and analyzed using a FAT-assembler to obtain interpretive sequence suitable for simple BLAST analyses. The inferred sequences were then analyzed using BLASTN against the non-redundant nucleotide database at NCBI to determine if the produced sequence would correctly determine the strains of FMDV tested by microarray. Additionally, the 10 best full genome alignments by score were obtained for performing phylogenetic analysis for comparison of predicted sequences to other closely related viral species members.

**Fig 3:**
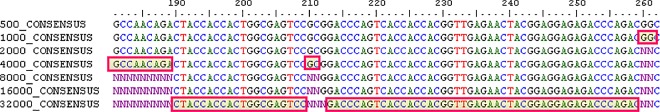
Consensus sequence buildup. Resolved sequence is selected by alignment of the candidate sequences for each of the thresholds. Sequence assembly is performed through selection of the individual resolved (non-ambiguous) nucleotides at the greatest MFI threshold.

Six of the 7 FMDV strains tested matched to the correct FMDV strain listed in Genbank with the highest BLAST score (Fig 3), demonstrating that the derived interpretive sequences had sufficient accuracy to identify the correct FMDV isolates. For FMDV Asia-1 Lebanon however, the matching Genbank FMDV strain was actually the second highest scoring sequence by BLAST with the first and second best scoring results coming from geospatially related FMDV Asia-1 viruses from Israel and Lebanon during the same outbreak year, 1989. These two isolates scored identical with regard to their alignment to the predicted microarray sequence (Max Score, Total Score, Query Coverage, E value, and Identity %) and nucleotide ratios.

From the results of the BLAST analysis, it does not appear that there is an obvious minimal threshold for percent coverage or identity in order to make a correct match. Retrospectively, it is not unexpected that the Asia-1 Lebanon isolate was co-identified with the FMDV Asia-1 Shamir isolate from Israel due to the mostly identical sequence of the two isolates. Thus it would be expected that problems for prediction would be more likely to occur between isolates of high similarity compared to more variable strains or genetic drift in culture. The accuracy of prediction will be highly dependent upon the quantity of sequence data in the database. For FMDV, which is under close surveillance due to its impact on agriculture and international trade, the database is relatively well defined. For emerging viruses with few sequences available in the NCBI database interpretation of sequence might be less effective. In such instances, direct sequencing or use of high density resequencing microarrays might be needed.

Phylogenetic analysis and comparison of the top 10 most closely related BLAST results show that highest scoring accession’s sequence matches the predicted sequence from the microarray. The caveat being as previously indicated, Asia-1, which shows that there is minimal distance between the Shamir and Lebanon strains. Based on results from the BLAST comparisons and alignments, it is expected that the differences between these two particular isolates are negligible, and may represent quasi-species from the same outbreak. (Table 1).

**Table 1:** Comparison of percent coverage and identity of microarray inferred interpretive sequences as compared to the predicted viral sequence record in Genbank. Sequence output from microarray was analyzed using the megaBLAST algorithm against Genbank. The identity of the highest scoring result was compared to the identity of the known viral FMD agents as confirmation of correct identification. Genbank accession numbers of the BLAST results (BLAST ID) are included for the expected nucleotide match, and the accession which identifies the sample sequence within Genbank (Sample ID) by the algorithm. All alignments were found to have significant E-scores of 0.0 by BLAST analysis.

FMDV Isolate	% Coverage	% Identity	Match	Sample Acc#	BLAST Acc# ID
A23 Kenya	100%	94%	YES	AY593766.1	AY593766.1
O3 Venezuela	96%	94%	YES	AY593827.1	AY593827.1
C	100%	97%	YES	AY593805.1	AY593805.1
Asia-1 Lebanon 989	100%	98%	NO*	AY593798.1	JF739177.1
SAT-1	97%	96%	YES	AY593846.1	AY593846.1
SAT-2	97%	81%	YES	AF540910.1	AF540910.1
SAT-3	97%	93%	YES	AY593850.1	AY593850.1

While gaps were observed in the predicted sequence, the amount of resolved sequence was sufficient for determining the most closely related isolate within Genbank. It is not unexpected that the analysis method is able to resolve ‘known’ agents by sequence, as the algorithm often selects the most closely related match during the template selection process. The analysis performs a sequence mapping very similar to assemblers currently used for resolving NGS data to known templates. However, the amount of data produced by microarray is obviously much less than that of NGS, with sequence variation resolved by substituting the MFI as a measure of confidence, as opposed to the occurrence of sequences within the overall population.

### Characterization of a Chimeric FMDV (O/A Chimera)

**Fig 4:**
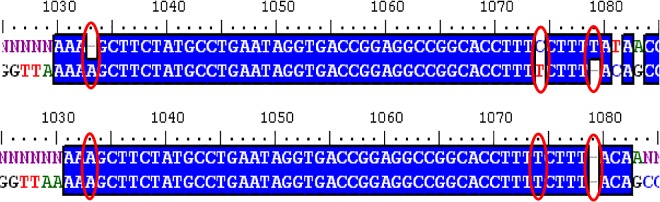
Comparison of specific misidentified nucleotides when comparing a chimeric virus to a “native template” (upper alignment) assembled sequence versus a “synthetic template” (lower alignment) assembled sequence to illustrate predicted indels and nucleotide error call improvement using the synthetic scaffold template.

A single chimeric isolate of FMDV, previously described by Rieder et al. [14], was obtained and tested by microarray. This chimeric virus strain is primarily of the O serotype with an A serotype derived insert. This analysis produced a resolved sequence of 95% identity to the original sequence as determined by Sanger sequencing methods. The resulting sequence was observed to have 65 incorrect base calls, and 48 unidentified nucleotides. It was observed that this method was better for correctly representing an insertion and deletion event, which was missed when using the single template (Fig 4). Additionally this information could be useful in the event of a forensics investigation for detecting engineered virus constructs by rapidly recognizing extensive localized variations within the sequence.

**Fig 5:**
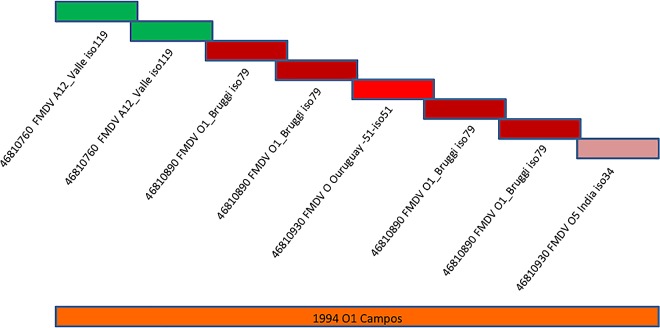
Synthetic template scaffold build-up compared to native template selected by the algorithm. This is the comparison of the components of the synthetic template buildup for a chimeric FMDV, compared to the selected template compared to an individual scaffold template selection.

Performing BLAST analysis on the output from the microarray is found to identify the O1 Campos isolate to be the best match with 100% coverage, and 96% identity. However, when looking at the alignment, the clear majority of errors are located in the first 720 nucleotides, representing the A12 insert within this chimera. Looking at the breakdown of the scaffold template, it is apparent that there are clearly two regions which have homology to distinct virus strains (Fig 5). Additionally, the phylogenetic analysis exhibited quite a bit of divergence from any of the known wild-type strains of FMDV.

### Effect of Mean Fluorescent Intensity (MFI) on Output Consensus Sequence

**Fig 6:**
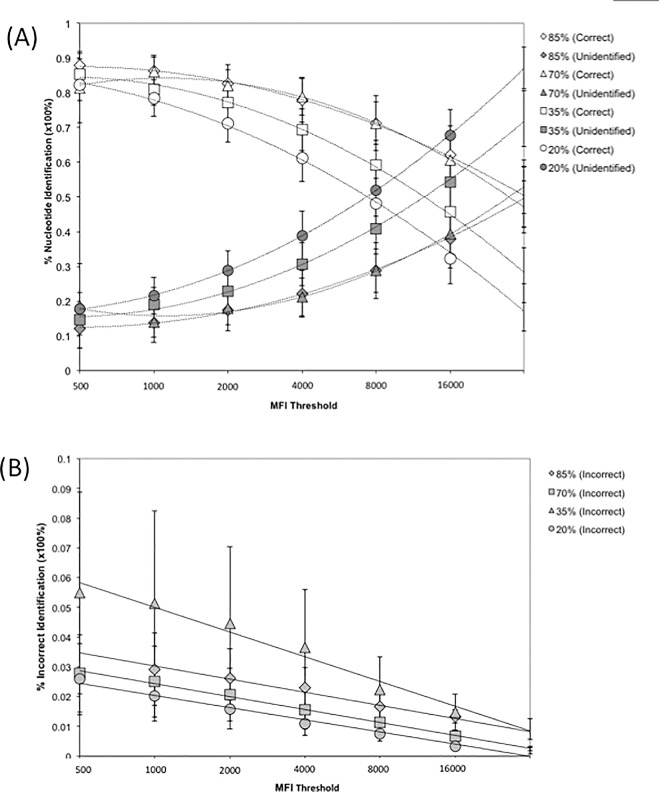
Effect of variable Mean Fluorescent Intensity (MFI) on the identification of nucleotides in the consensus output. (A) △ 85% Power (Correct); ▲ 85% Power (Unidentified); ◇ 70% Power (Correct); ◆ 70% Power (Unidentified); □ 35% Power (Correct); ■ 35% Power (Unidentified); ○ 20% Power (Correct); ● 20% Power (Unidentified). Decreasing average MFI results in an overall reduction of correct nucleotides with an inverse increase in ambiguous nucleotides; seen as a shift in the 50% correct:unidentified ratio to a lower threshold. (B) ▲ 85% Power (Incorrect); ◆ 70% Power (Incorrect); ■ 35% Power (Incorrect); ● 20% Power (Incorrect) Effect of variable MFI on the percent of incorrectly identified nucleotides at different threshold cutoffs.

To evaluate the potential effects of reduced specific fluorescent intensity due to variability in nucleic acid quantity or quality on the final consensus sequence, we repeated the analysis using varied laser intensities (20%, 35%, 70%, and 85% power). The percentages of correctly identified nucleotides were contrasted to unidentified nucleotides over the range of minimal thresholds to determine the effectiveness of the prediction algorithm at varying parameters (Fig 6A). Additionally, incorrectly identified nucleotides were plotted along the same axis to determine the increase in predictive errors under the various conditions.

We observed that the number of nucleotides that were considered ‘unidentified’ were increased and the nucleotides identified as ‘correct’ were decreased as a result of the reduction in MFI. While, increased laser power does result in more overall ‘correct’ nucleotide calls; we also observed that lower thresholds with increased laser power demonstrated an increase in nucleotides being ‘incorrectly’ identified. Even with this increase in the error rate, at the lowest threshold, the sequence mean identification was between 80%-90% correct, with <20% unidentified and between 2%-6% incorrect (Fig 6B); thus indicating a relatively high degree of resolution for sequence identification regardless of the average MFI. From this we determine that the overall effect of reduced MFI is a slight increase in ambiguity with the reduction in fluorescence, but no loss of sequence specificity.

While this method of analysis is certainly useful for analyzing genotyping microarrays, it may also have utility in analysis of high density resequencing microarrays as well as NGS applications as a mechanism for producing scaffolds for building the alignments. The difficulty with this is performing multiple population analyses on the large amount of NGS data in order to produce the initial synthetic template.

While the microarray method is not necessarily a better approach than NGS, it does have some advantages in specific situations, and also is expected to produce results in a shorter time span than would be expected from most current NGS technologies. The microarray library preparation can function with smaller products, while many NGS technologies require larger nucleotide strands for library preparation. This means that samples with degraded RNA may be more likely to be resolved using the microarray method than current NGS protocols. The microarray is also more rapid, and doesn’t require barcoding for multiplexing, as each individual sample is physically sequestered upon the microarray slide. While not as practical for a research setting, this technology would be considered more useful in a diagnostic setting for rapid identification, in lieu of PCR and Sanger sequencing.

Genotyping microarrays are useful tools for characterization of viruses in the event of an outbreak such as FMD which high degrees of genomic polymorphism between serotypes and species. As the density of features on microarrays continues to increase, the potential for improved identification will also expand. However, even with high-density arrays, efficient algorithms for feature selection and analysis are still necessary, and will further increase the power of these platforms. Here we describe an FMD serotyping microarray, as well as an algorithm for genomic sequence prediction.

The genotyping microarray, when combined with methods of random amplification, is a powerful, relatively simple tool for many applications including diagnostics. In this application, we describe a microarray which can be utilized to identify and characterize isolates of FMDV. This genotyping approach has advantages over traditional sequence analysis because it does not require specific sequencing primers to identify the correct serotype. As the system relies on random amplification, regions of sequence are able to be identified rapidly and used as genomic ‘fingerprints’ to assist in virus characterization. This work also illustrates the importance of efficient algorithms for analysis that can further stretch the utility of genotyping microarrays without the need for major design improvements to the arrays themselves.
